# Sensitivity of Global and Regional Terrestrial Carbon Storage to the Direct CO_2_ Effect and Climate Change Based on the CMIP5 Model Intercomparison

**DOI:** 10.1371/journal.pone.0095282

**Published:** 2014-04-18

**Authors:** Jing Peng, Li Dan, Mei Huang

**Affiliations:** 1 START Temperate East Asia Regional Center and Key Laboratory of Regional Climate-Environment for Temperate East Asia, Institute of Atmospheric Physics, Chinese Academy of Sciences, Beijing, China; 2 Key Laboratory of Ecosystem Network Observation and Modeling, Institute of Geographical Sciences and Natural Resources Research, Chinese Academy of Sciences, Beijing, China; Centro de Investigacion Cientifica y Educacion Superior de Ensenada, Mexico

## Abstract

Global and regional land carbon storage has been significantly affected by increasing atmospheric CO_2_ concentration and climate change. Based on fully coupled climate-carbon-cycle simulations from the Coupled Model Intercomparison Project Phase 5 (CMIP5), we investigate sensitivities of land carbon storage to rising atmospheric CO_2_ concentration and climate change over the world and 21 regions during the 130 years. Overall, the simulations suggest that consistently spatial positive effects of the increasing CO_2_ concentrations on land carbon storage are expressed with a multi-model averaged value of 1.04PgC per ppm. The stronger positive values are mainly located in the broad areas of temperate and tropical forest, especially in Amazon basin and western Africa. However, large heterogeneity distributed for sensitivities of land carbon storage to climate change. Climate change causes decrease in land carbon storage in most tropics and the Southern Hemisphere. In these regions, decrease in soil moisture (MRSO) and enhanced drought somewhat contribute to such a decrease accompanied with rising temperature. Conversely, an increase in land carbon storage has been observed in high latitude and altitude regions (e.g., northern Asia and Tibet). The model simulations also suggest that global negative impacts of climate change on land carbon storage are predominantly attributed to decrease in land carbon storage in tropics. Although current warming can lead to an increase in land storage of high latitudes of Northern Hemisphere due to elevated vegetation growth, a risk of exacerbated future climate change may be induced due to release of carbon from tropics.

## Introduction

Variations in the whole terrestrial carbon cycle are accompanied with increasing atmospheric CO_2_ concentration and climate change. The physical climate can influence the terrestrial carbon storage and the carbon exchange between atmosphere and land [1]–[5], and the sequent changes in atmospheric concentration of CO_2_ simultaneously affect the physical climate system [1], [6]. By the end of the twenty-first century, there is an additional CO_2_ change between 20 and 200 ppm considering warming alone, while the higher CO_2_ values can lead to an additional climate warming ranging between 0.1° and 1.5°C [1]. Zeng et al. (2004) [7] also suggested a positive feedback to global warming from the interactive carbon cycle introduces an additional increase of 90 ppm in the atmospheric CO_2_ and then 0.6 degree additional warming during the period of 1860–2100 with the prescribed IPCC-SRES-A1B emission scenario. Regionally, using the Coupled Climate Carbon Cycle Model Intercomparison Project (C^4^MIP), Cox et al. (2013) [8] estimates that warming alone will release 53±17 gigatonnes K^−1^of carbon to the atmosphere over tropical land from 30° north to 30° south. On the other side, increasing CO_2_ concentrations would influence land carbon balance, such as the stimulation of carbon storage, increases in gross primary production, net primary productivity and heterotrophic respiration [6], [8]–[12]. With increasing CO_2_ concentrations alone there is a widely distributed terrestrial carbon sink of 1.4–3.8 PgCyr^−1^ during the 1990s, rising to 3.7–8.6 PgCyr^−1^ a century later [13]. Such an enhancement of land carbon storage was accessed due to CO_2_ fertilization effects with a rate of 0.07PgCyr^−1^ using simulation from Organizing Carbon and Hydrology considering fire disturbance [12].

As sensitivities of land carbon storage are largely disturbed and altered by both increasing CO_2_ concentrations and climate change [12], [14]–[18], previous studies have focused on it [1], [14]. Generally, when climate change is only accounted for, a reduction is suggested for the efficiency of the terrestrial ecosystems to absorb anthropogenic carbon emissions [2], [19], [20] and general negative sensitivities of land carbon storage have been revealed from the earth system models (ESMs)[14] and Dynamic Global Vegetation Models (DGVMs) [19]. Arora et al. (2013) [14] estimated earth system models (ESMs) simulations from CMIP5, which represent the interactions between the carbon cycle and physical climate system. Negative impacts of continuous warming on terrestrial carbon storage have been suggested. Sitch et al. (2008) [19] used the five Dynamic Global Vegetation Models (DGVMs) and IPSL GCM to evaluate the terrestrial carbon cycle and climate-carbon cycle feedbacks, and found the similar sign of sensitivities of terrestrial land carbon to the changes in atmospheric CO_2_ and climate. Generally, terrestrial carbon balance and storage is discovered to be sensitive to rising atmospheric CO_2_ concentration and climate change [17], [21], [22]. Land carbon sensitivities to rising CO_2_ are positive implying increases under enhanced atmospheric CO_2_ concentration about 1.19∼1.32 PgC ppm^−1^, while its sensitivities to temperature are negative ranging between −137∼−85 PgCK^−1^ [23].

Although both the common results suggest the positive response of terrestrial carbon storage to increasing CO_2_ concentration and its negative response to climate change [1], [8], [23], many potentially uncertainties are remained [14], [19], such as the response magnitudes and regional pattern, often because they are poorly understood [24], or scantily quantified at scales relevant for models [1]. Several previous studies have been carried in order to evaluate and predict land carbon sensitivities to CO_2_ and temperature [1], [14], [23]. But most of them have not distinguished the regional variability due to impacts of rising atmospheric CO_2_ concentration or climate change on carbon storage in order to identify the sensitive regions, respectively. On the basis of this point, we focus on the sensitivities of land carbon storage to the rising atmospheric CO_2_ concentrations and climate change at global and regional scales by multi-models, and compare the simulated variations between models that represent a potential source of uncertainty. Among regions which are more fragile to climate change, we quantify sensitivities of land carbon storage at regional scales. It is important for mitigation and adaptation of terrestrial ecosystems to climate change. In this study, we use simulations from earth system models offered by the CMIP5 to quantify and compare the carbon sensitivities to changes in CO_2_ and climate for the period from 1860 to 1989. The disturbances of none-CO_2_ greenhouse gases, land use and fire are totally ignored.

## Experiments and Methods

The fifth phase of Coupled Model Intercomparison Project (CMIP5) can provide a common framework [25] to compare and assess land carbon storage responses in the context of climate simulations [14]. There are two experiments used in this analysis, which have been downloaded from http://cmip100pcmdi.llnl.gov/cmip5/forcing.html. These chosen experiments from CMIP5 have been described by Peng et al.(2013; 2014) [26], [27]. They are detailed as follows: (1) in the experiment of only considering the single effect of atmospheric increasing CO_2_ concentrations, biogeochemistry only responds to the increasing CO_2_ concentrations in land models while the radiative forcing is fixed at the pre-industrial values in the atmospheric modules; (2) in the experiment of only thinking of the single effect of climate change, the radiative forcing responds to the increasing atmospheric CO_2_ concentration (prescribed by 1% increase of CO_2_ concentration per year in atmosphere from the pre-industrial values to the quadruple) while the biogeochemistry remains at the pre-industrial values in the biogeochemistry modules. In addition, in order to estimate and compare the sensitivities of land carbon storage to increasing CO_2_ concentration and climate change, we use the simulations from six of the fully carbon-climate coupled ESMs, which participate in the CMIP5 intercomparison project. These models include HadGEM2-ES [28], IPSL-CM5A-LR [29], CESM1-BGC [14], MPI-ESM-LR [30], [31], CanESM2 [32] and BCC-CSM1-1 [33].

The ESMs, which are used to analyze the sensitivities, except for HadGEM2-ES and MPI-ESM-LR, do not include dynamical vegetation cover. Their spatial distribution is controlled by the competition between different PFTs (Plant functional types) [14]. A patch-based representation of vegetation structure competition has been made [34]. HadGEM2-ES can simulate the transient shifts and geographic patterns of vegetation. Its atmosphere/land component resolution is 1.6° by 1.6°. A mixture of 12 PFTs is shown in HadGEM2-ES [28]. Over each patch a transient dynamics of vegetation also has be presented by MPI-ESM-LR. It represents the resolution of atmospheric component of 1.9° by 1.9° [31].

The IPSL-CM5A-LR [29] is the new generation Earth System Model developed by the Institute Pierre Simon Laplace. A resolution of 3.6° by 1.8° at latitudes and longitudes is used in the land and atmospheric components. There are 39 vertical levels of atmosphere. With a daily time step land component ORCHIDEE [35] simulates processes of photosynthesis, carbon allocation, litter decomposition, soil carbon dynamics, maintenance and growth respiration and phenology for 13 different plant functional types [34].

Version 1 of the Community Earth System Model (CESM1) is the successor to version 4 of the Community Climate System Model (CCSM4). In this model, global climate model consisting of land, atmosphere, ocean, and sea-ice components has been fully coupled [36].The terrestrial carbon cycle is represented by the CLM4 land surface scheme. And it contains 16 different PFTs. Coupled carbon-nitrogen dynamics are included and expressed as CLM4CN [37], [38].

CanESM2 is the new generation Earth System Model developed from the first generation Canadian earth system model (CanESM1) [21], [39]. It is produced and developed by the Canadian Centre for Climate Modelling and Analysis (CCCma) and described by Arora et al. (2013) [14]. The atmospheric component has horizontal resolution of about 2.8° in a grid cell. The Canadian Terrestrial Ecosystem Model (CTEM) [40] is used to model terrestrial ecosystem processes. It simulates terrestrial carbon supported by three live vegetation pools (leaves, stem, and root) and two dead pools (litter and soil organic carbon) for PFTs (e.g. needle leaf evergreen and deciduous trees, broadleaf evergreen and cold and dry deciduous trees, and C3 and C4 crops and grasses) [14].

BCC-CSM1.1 is a fully coupled global climate–carbon model including interactive vegetation and global carbon cycle [33]. The atmospheric component BCC-AGCM2.1 is a global spectral model with a horizontal resolution of 2.81 degrees, with vertical 26 levels of atmosphere. For the land surface processes, the land surface component Atmosphere-Vegetation Interaction Model (AVIM) [41], [42] is incorporated into the biogeophysical frame of NCAR/CLM3. With 15 PFTs including natural vegetation and crop, each a grid cell can contains up to four PFTs. Terrestrial carbon cycle components simulates biochemical and physiological processes. For example, photosynthesis and respiration of vegetation, allocation of carbohydrate to leaves, stem, and root tissues, carbon loss due to turnover and mortality of vegetation can be modeled as described by previous work [43].

**Figure 1:**
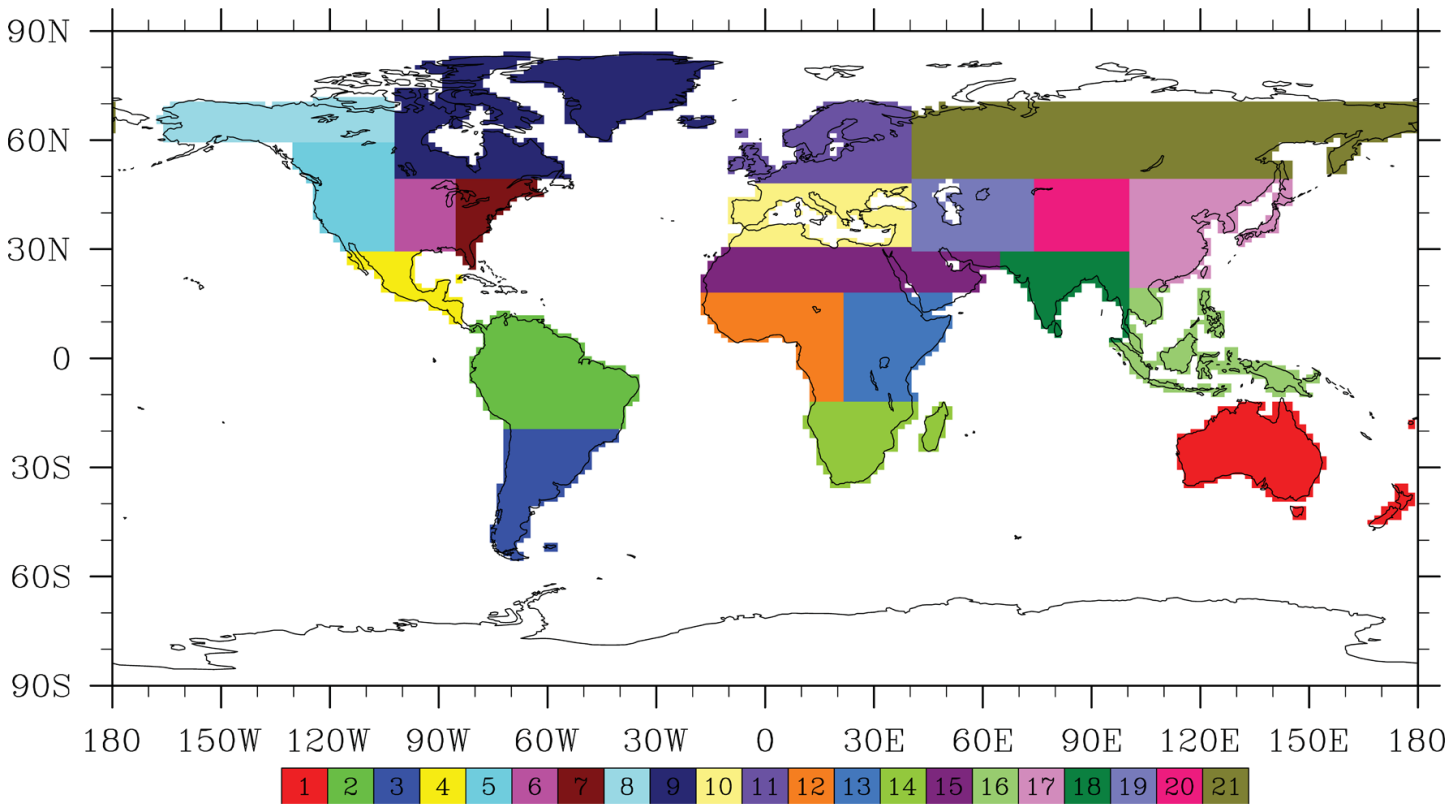
Map of 21 regions.

In order to analyze the outputs simulated by the global coupled models, a criteria of regions has been employed by Peng et al. (2013; 2014)[26],[27] in the regional scale performance (Table 1 and Figure 1). It has been described by Giorgi et al (2000) [44] as follows: (1) the size of the regions vary in the range of a few thousand to several thousand km in each direction, so that each region includes at least a few Earth system models (ESM) grid points and thus contains the smallest wavelength of ESM solutions; (2) all land areas in the World with a number of regional management simple shapes have been approximately covered; (3) selection of specific regions is in order to represent different climatic regimes and terrain settings. On the basis of such criteria of regional selection, we calculate the sensitivities of global and regional land carbon storage to climate change and increasing atmospheric CO_2_ concentrations. These sensitivities to direct CO_2_ effects and climate change are described as 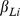 (Pg C per ppm) and 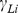 (Pg C K^−1^) as the following equations, respectively. The calculation method can be referred to a previous study reported by Cox et al. (2013) [8].
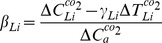
(1)

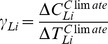
(2)


**Table 1:** List of 21 regions.

ID	Region	Abbreviation
1	Australia	AUS
2	Amazon basin	AMZ
3	southern South America	SSA
4	Central America	CAM
5	western North America	WNA
6	central North America	CNA
7	eastern North America	ENA
8	Alaska	ALA
9	Greenland	GRL
10	Mediterranean basin	MED
11	northern Europe	NEU
12	western Africa	WAF
13	eastern Africa	EAF
14	southern Africa	SAF
15	Sahara	SAH
16	southeastern Asia	SEA
17	eastern Asia	EAS
18	southern Asia	SAS
19	central Asia	CAS
20	Tibet	TIB
21	northern Asia	NAS

Where 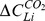 is the change in global or regional land carbon storage (in PgC), 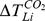
is the change in mean global and regional atmospheric temperature (in K) and 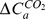
is the change in atmospheric CO_2_ concentration (in ppm), in response to increasing atmospheric CO_2_ concentrations. 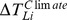
is the change in mean global and regional atmospheric temperature (in K) and 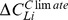 is the change in global and regional land carbon storage (in PgC), in response to climate change. These changes in temperature, land carbon storage and atmospheric CO_2_ concentration averaged over the period of the last 30 years of 130-years relative to the period of the first 30 years in all cases of calculation for the sensitivities responding the increasing CO_2_ concentration (γ_Li_) and climate change (β_Li_).

In this analysis, R is a correlation coefficient between the annual variable (e.g., temperature, precipitation) and the natural sequence 1, 2, 3,…, n, at a given year [45]. R is calculated by the following formula:
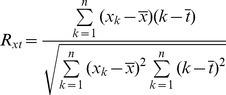
(3)


Where x_k_ is the annual variable in the time k, n is the sequential year, and 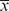 is the multi-year mean for this variable; 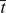 is equal to the mean of 1 and n. The linear trend of the variable is presented within the period of 1860–1989. When a “significant” linear trend for a variable is shown, R must pass the significance level (e.g., P<0.05) using the student t-test.

## Results

**Figure 2:**
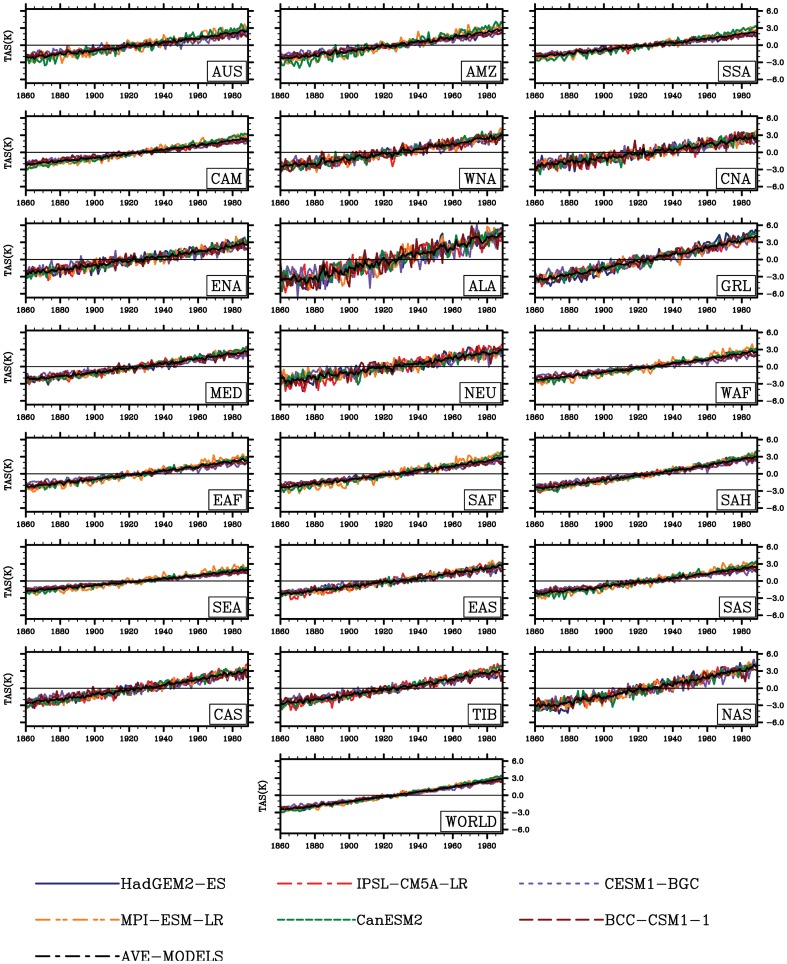
Variability in anomalies of annual mean surface air temperature (K) at regional and global scales considering climate change alone.

**Figure 3:**
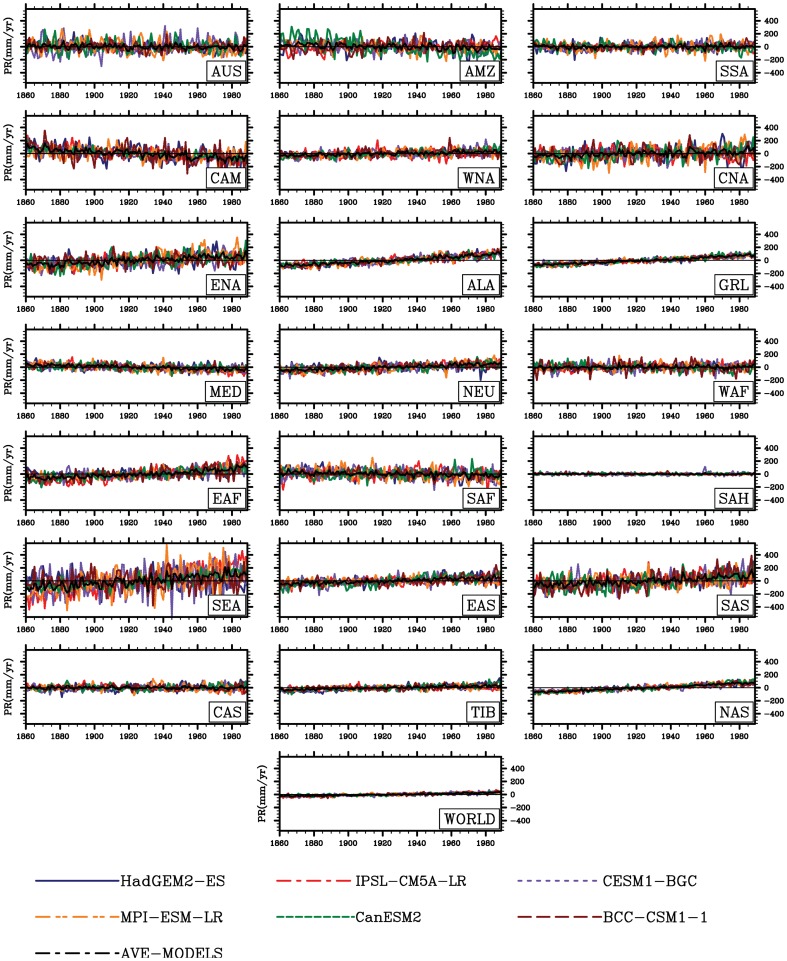
Same as Figure 2, but for annual precipitation (mm yr^−1^).

**Figure 4:**
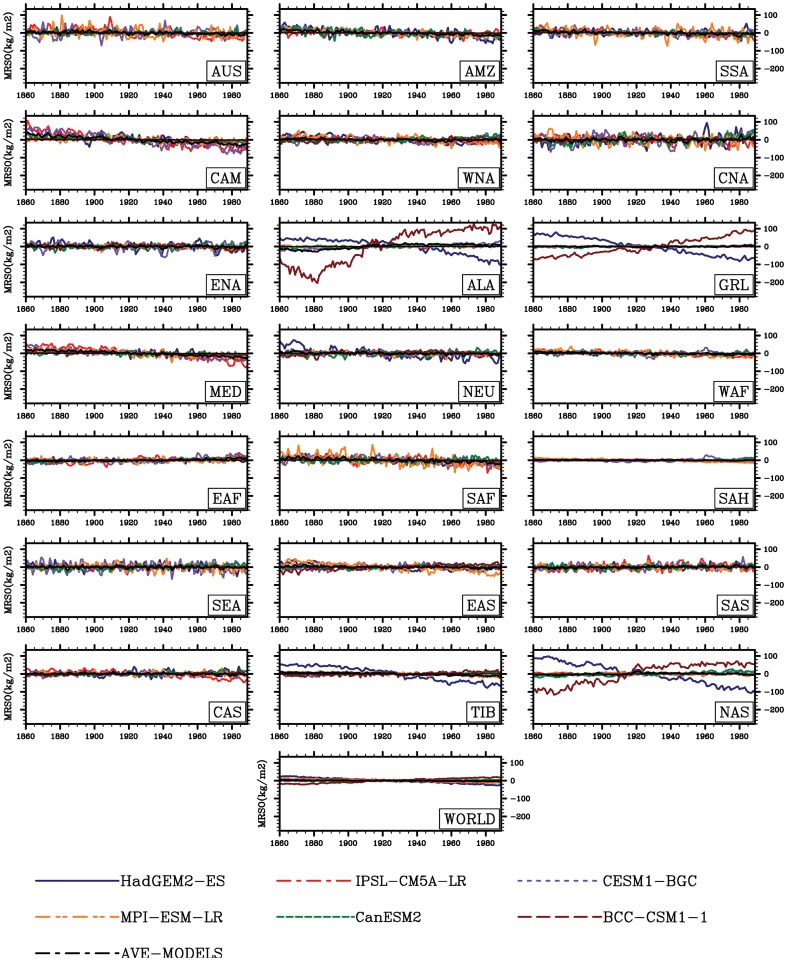
Same as Figure 2, but for soil moisture (kg m^−2^).

The global and regional climate consistently show increasing trends in temperature (0.05 °C yr^−1^, R = 0.95, P<0.001), but no consensus trends in precipitation (PR) and soil moisture are presented at regional scales (Figures 2–4). As shown in Figure 3, increases in precipitation in high-latitude regions are shown (e.g., in northern Asia with 1.17mm yr^−2^, R = 0.97, P<0.01), while decreases in precipitation are located in part of the low latitudes such as Amazon basin with −0.50mm yr^−2^ (R = 0.50, P<0.01) and Central America with −1.28 mmyr^−2^ (R = 0.79, P<0.01). These spatial divergences on changes in soil moisture are also shown in Figure 4. Only considering climate change, the increases in soil moisture is mainly distributed in high latitudes, while the area principally in Amazon basin exhibits a considerable decrease of soil moisture with −0.23 kg yr^−1^ (R = 0.86, P<0.001).

**Figure 5:**
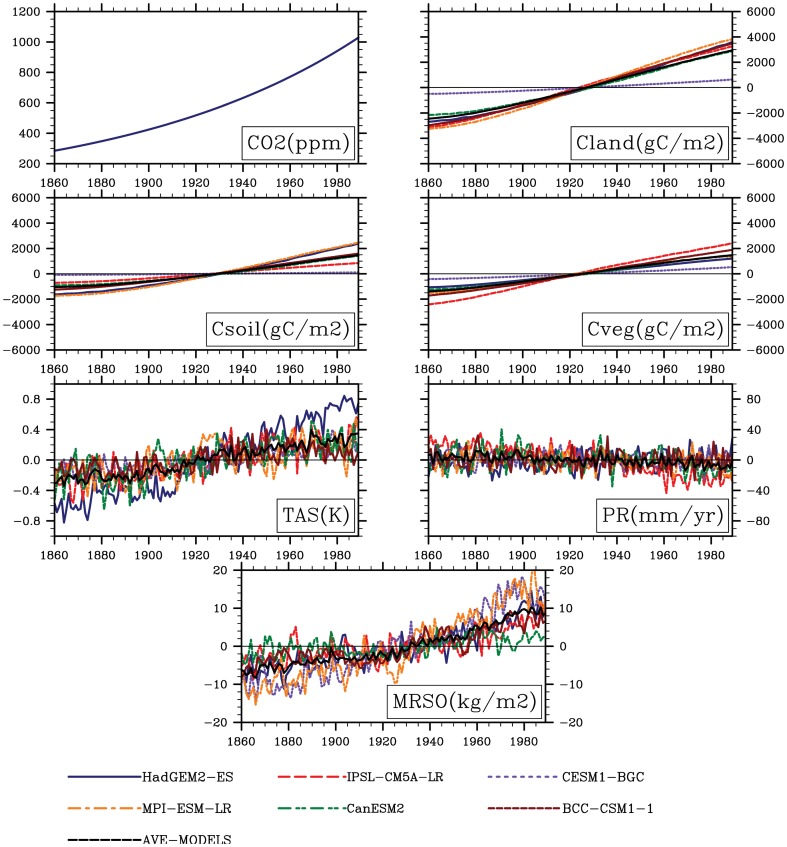
Variability in atmospheric CO_2_ concentration (ppm) at rate of 1% per year from pre-industrial values until concentration quadruples for a 130-year long simulation. variability in anomalies of global land carbon storage (Cland, gC m^−2^), soil carbon storage (Csoil, gC m^−2^), vegetation carbon storage (Cveg, gC m^−2^), annual mean temperature (TAS, K), annual precipitation (PR, mm yr^−1^) and soil moisture (MRSO, kg m^−2^) only allowing for the direct CO_2_ effect.

Figure 5 shows the interannual variability in modeled global annual land carbon storage anomaly, soil carbon storage anomaly, vegetation carbon storage anomaly, mean temperature anomaly, annual precipitation anomaly and soil moisture anomaly only responding to the CO_2_ increases. As a result, the increase in global annual land carbon storage has been presented across the whole globe. Such an increase in turn affects the soil carbon storage with increasing rate of 20.7 gC yr^−1^ (R = 0.989, P<0.001).

**Figure 6:**
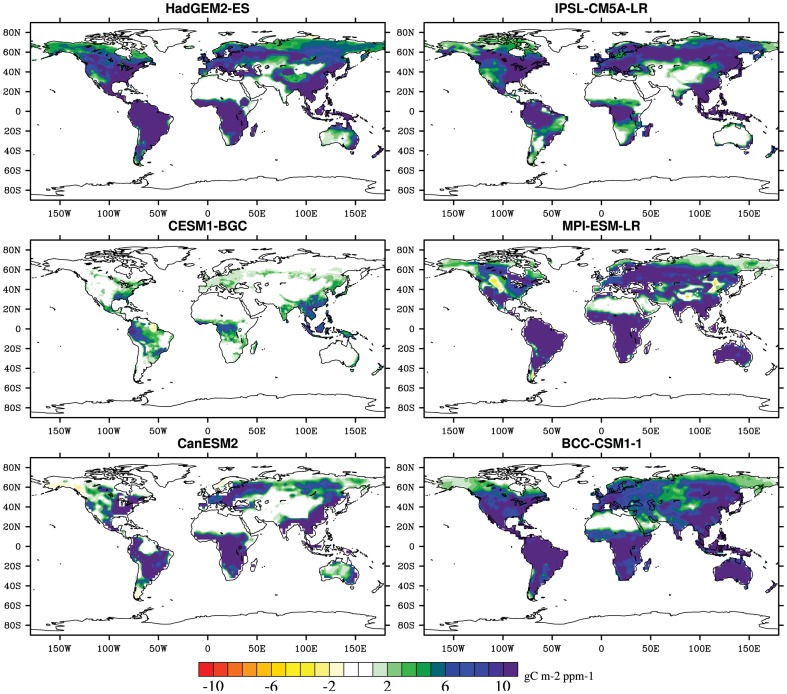
Spatial distribution in sensitivity of terrestrial carbon storage to upward atmospheric CO_2_ concentration simulated from six the fifth Coupled Model Intercomparison Project (CMIP5) models. units: gC m^−2^ ppm^−1^.

**Figure 7:**
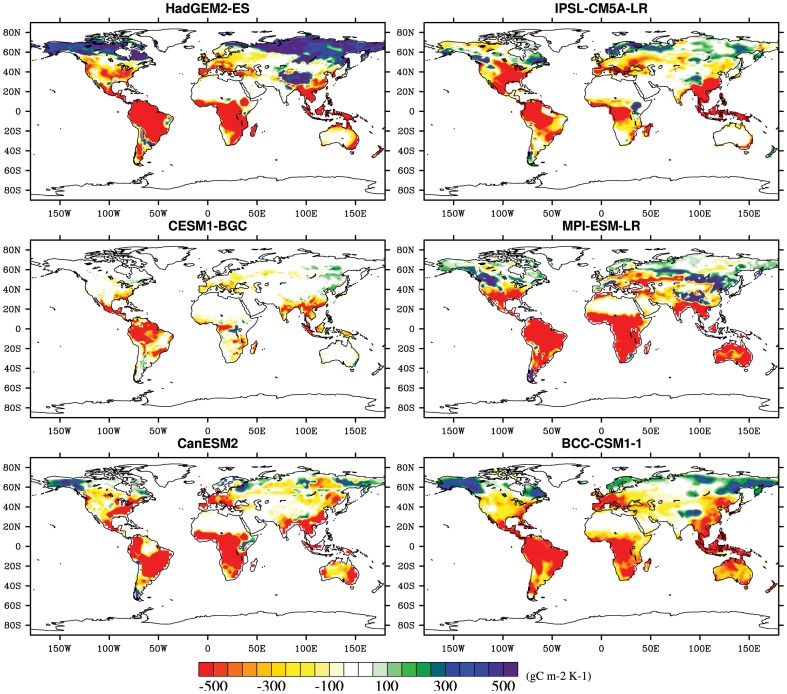
Same as Figure 9, but to the warming (gC m^−2^ K^−1^).

The geographical distribution of in the direct CO_2_ impacts on the terrestrial and regional land carbon storage is shown in Figures 6 and Table 2. Sensitivities of land carbon storage, considering the CO_2_ fertilization effect alone, are fairly positive in the most terrestrial ecosystem, which means the terrestrial biosphere acting as enhanced carbon storage by 1.0 Pg C per ppm. The most dramatic increases caused by the increased CO_2_ concentrations are mostly located in the regions such as Amazon basin, northern Asia, western Africa and eastern Africa, which have larger vegetation biomass than other regions. However, the warming greatly reduces the land carbon storage and the carbon sequestration. Excluding Tibet, northern Asia, Alaska and Greenland, the impacts of the warming on land carbon storage are negative in the terrestrial ecosystems with about −41.6 PgCK^−1^. Additionally, it should be noted that these simulations forced by both increasing CO_2_ concentration and climate change are properly different among six ESMs and regions. The multi-model values across the globe range from 0.2 PgC per ppm in CESM1-BGC to 1.4 PgC per ppm in MPI-ESM-LR responding the direct CO_2_ effects and from −17.5 PgC K^−1^ in CESM1-BGC to −58.6 PgC K^−1^ in CanESM2 considering climate change alone across the whole terrestrial ecosystem (Tables 2–3 and Figures 6–7).

**Table 2:** The impact of increasing atmospheric CO_2_ concentration on global and regional land carbon storage (units: PgC ppm^−1^).

ID	Acronym	HadGEM2-ES	IPSL-CM5A-LR	CESM1-BGC	MPI-ESM-LR	CanESM2	BCC-CSM1-1
1	AUS	0.04	0.02	0.01	0.1	0.04	0.1
2	AMZ	0.22	0.19	0.04	0.24	0.12	0.21
3	SSA	0.08	0.03	0.01	0.06	0.06	0.06
4	CAM	0.04	0.02	0.01	0.03	0.02	0.03
5	WNA	0.04	0.04	0	0.03	0.02	0.05
6	CNA	0.04	0.04	0.01	0.02	0.03	0.03
7	ENA	0.03	0.03	0.01	0.02	0.05	0.03
8	ALA	0.02	0.01	0	0.01	0	0.01
9	GRL	0.02	0.04	0	0.02	0.01	0.01
10	MED	0.03	0.03	0	0.05	0.03	0.03
11	NEU	0.04	0.05	0	0.04	0.03	0.03
12	WAF	0.09	0.08	0.02	0.12	0.12	0.08
13	EAF	0.09	0.06	0.01	0.14	0.11	0.1
14	SAF	0.09	0.03	0.01	0.12	0.08	0.06
15	SAH	0	0	0	0.01	0	0.01
16	SEA	0.07	0.13	0.03	0.09	0.13	0.08
17	EAS	0.09	0.08	0.03	0.06	0.1	0.09
18	SAS	0.04	0.04	0.02	0.07	0.07	0.05
19	CAS	0.02	0.01	0	0.06	0.01	0.04
20	TIB	0.03	0.01	0	0.03	0	0.03
21	NAS	0.1	0.15	0.01	0.1	0.06	0.09
22	WLD	1.21	1.08	0.21	1.42	1.09	1.23

**Table 3:** The impact of rising temperature on global and regional land carbon storage (units: PgCK^-1^).

ID	Acronym	HadGEM2-ES	IPSL-CM5A-LR	CESM1-BGC	MPI-ESM-LR	CanESM2	BCC-CSM1-1
1	AUS	−1.93	−0.99	−0.19	−6.25	−3.42	−3.15
2	AMZ	−13.88	−9.99	-7.45	−12.85	−10.58	−8.49
3	SSA	−2.78	−1.25	−0.84	−2.61	−3.85	−2.48
4	CAM	−2.04	−1.53	−1.49	−1.9	−2.01	−1.52
5	WNA	−1.03	−0.6	−0.1	−0.27	−1.13	−0.54
6	CNA	−0.86	−2.64	−0.33	−1.53	−1.15	−0.74
7	ENA	−0.57	−0.79	−0.29	−0.49	−1.62	−0.83
8	ALA	1.51	−0.35	0.01	0.19	0.55	0.61
9	GRL	1.44	0.39	−0.02	0.39	0.11	0.33
10	MED	−1.37	−1.62	−0.56	−2.01	−2.14	−1.51
11	NEU	−0.59	−0.35	−0.19	−0.76	−1	−0.71
12	WAF	−4.38	−3.14	−0.97	−6.17	−7.61	−3.82
13	EAF	−4.71	−0.97	−0.22	−6.22	−4.27	−3.18
14	SAF	−4.67	−1.61	−0.88	−6.21	−4.94	−2.72
15	SAH	−0.03	0	−0.01	−0.37	−0.07	−0.24
16	SEA	−5.26	−5.13	−1.28	−3.9	−6.54	−3.45
17	EAS	−1.29	−3.05	−0.85	0.45	−3.74	−2.93
18	SAS	−0.86	−1.85	−1.44	−3.19	−3.53	−0.75
19	CAS	−0.4	−0.49	−0.1	−1.9	−0.29	−0.84
20	TIB	1.65	0.07	−0.01	0.67	−0.3	0.21
21	NAS	4.94	0.09	−0.05	0.97	−0.66	1.24
22	WLD	−36.52	−35.92	−17.46	−53.78	−58.59	−35.49

**Figure 8:**
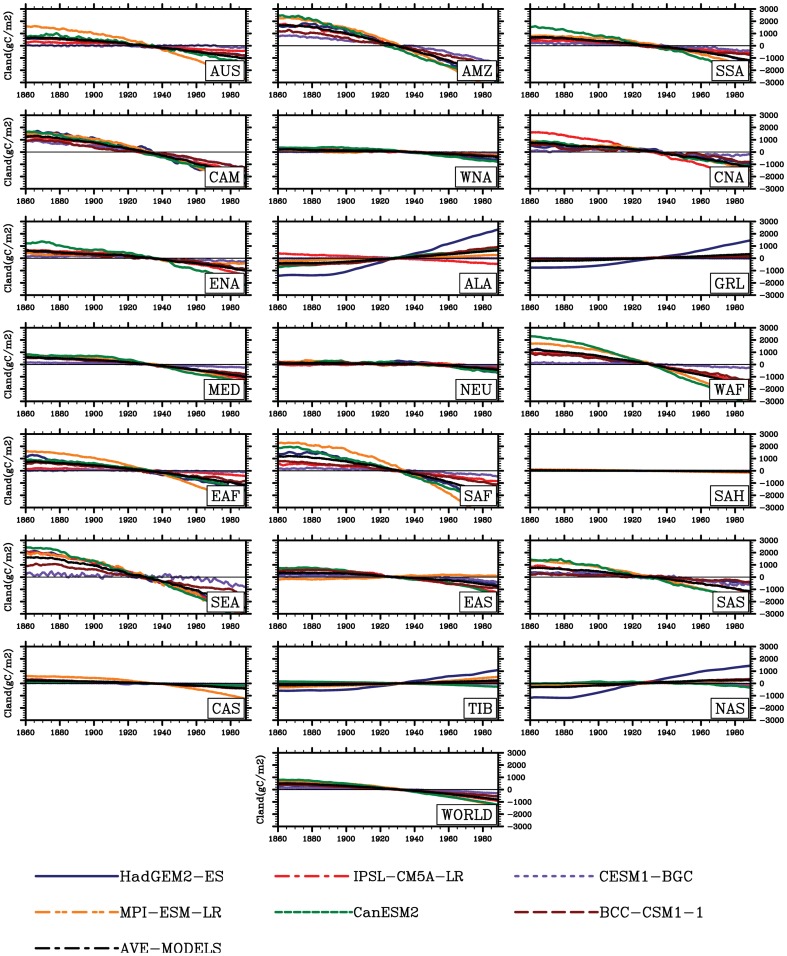
Same as Figure 2, but for land carbon storage (Cland, gC m^−2^).

Figure 7 exhibits the spatial pattern of changes in land carbon storage only responding to warming. In response to such a warming, the largest loss in land carbon storage is mainly distributed in central Amazon basin, southeastern Asia, Central America and eastern Africa (Table 3 and Figure 7). Generally, negative sensitivities of land carbon storage mostly located in the tropics and Southern Hemisphere. Inversely, areas in northern high latitudes primarily exhibit a considerable increase of land carbon storage. For example, a significantly enhanced carbon storage appears in Tibet with 3.26 gC m^−2^ yr^−1^ (R = 0.98, P<0.001). Increases in land carbon storage in northern Asia and Greenland Alaska are also detected (Figure 8). The simulation shows a consistent pattern (a negative sensitivity for the land carbon storage to temperature across the major land) modeled by the major models in terms of the sign of sensitivities of land carbon storage to temperature. Spatial discrepancies among regions are evidently presented as mentioned above: increases in land carbon storage in the mainly temperature-limited regions, but decrease in higher temperature regions. On the other side, in comparison with different regions in the Figures 7, it's worth noting that the multi-model simulations provide fairly consistent results in most tropical areas and Southern Hemisphere (e.g. Australia, Eastern Africa, Southern Africa, Western Africa, Southeast Asia, Amazon Basin), but inconsistent results in a part of northern latitudes (e.g. some show positive values; others show negative values). The inconsistency is particularly evident in high latitudes of Northern Hemisphere, such as Alaska and part of northern Europe.

**Figure 9:**
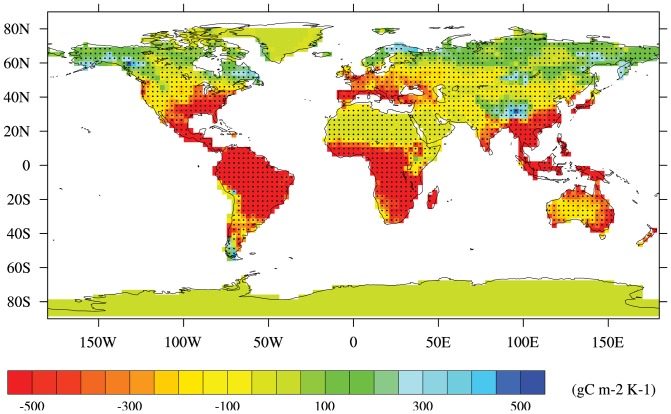
Six model average of the sensitivities of land carbon storage to climate change and agreement between simulations. Hatched areas means 4 or more of models agree on the same sign in the change in sensitivities to the rising temperature.

Spatially, analysis of responses of land carbon storage to climate change also reveals major pattern: negative impacts of rising temperature at the low and middle latitudes of the World are simulated by the major models in terms of the sign of response of land carbon storage; it's positive impacts at the high latitudes is presented. The changes are defined as averaged values of the period 1960–1989 relative to 1860–1889 in order to calculate these sensitivities as mentioned above. In Figure 9, in response to rising atmospheric temperature, average zonal-annual (sensitivities averaged over the regions and year) patterns and the agreement between simulations are deduced: ≥80% of areas across the whole terrestrial ecosystems shows the same sign of land carbon storage sensitivities modeled by a majority of simulations (≥4/6 simulations). A majority of the simulations agrees on the negative impact of temperature in tropics and positive impact in temperature-limited regions in response to climate change. For example, 99.6% of Amazon basin and 99.2% of western Africa show the negative impacts of increasing temperature on the land carbon storage. In contrast, the reverse sensitivities to warming are shown both quantitatively and qualitatively in high latitudes of Northern Hemisphere. In the most areas, there is little agreement among simulations for the sensitivities to increasing temperature around 60°N and Greenland.

## Discussion

In terms of regions (Figures 2–4), the spatial patterns of temperature, precipitation and soil moisture are so different. In high latitudes and altitudes, temperature and radiation impose a complex and varying limitations on vegetation activity [60]. For example, in the Western Europe, solar radiation is an evident limited factor to the vegetation growth. In the eastern of Tibetan plateau temperature change is the main cause to affect the vegetation growth and NPP [47]. Accompanied with such warming, increases in soil moisture have been reported by Peng et al. (2013) [26] in these regions. However, the tropical change estimated from the simulations is currently different from that of temperature-limited regions. Indeed water limitation is enhanced (e.g. increased droughts) in some of these tropical regions. Regionally, the CO_2_-induced climatic change enhances a decrease in soil moisture most in Amazon basin and part of arid and semi-arid regions (e.g. Mediterranean basin, Sahara and central Asia). In some cases, only considering the radiative CO_2_ forcing, the increasing atmospheric CO_2_ can influence the climate factors (e.g., rising temperature). Although the increasing atmospheric CO_2_ only influences the climate but not the biogeochemistry, the climatic change in turn affects the biogeochemistry. It can be to say biogeochemistry can respond to rising temperature and changes in other climatic variables. For example, associated warming, the increased evapotranspiration produced by increasing temperature can also exacerbate drought in Amazon basin [48]. As a consequence, enhanced drought can cause reduction in captured carbon in living biomass.

There is current lack of information on the accurate magnitude of the response of terrestrial carbon storage and the affecting causes, and thus large differences exist among different ESMs. Such differences among models are determined by the used approaches (e.g., the models using a biogeochemical approach to calculate the terrestrial photosynthesis) [14], which provides an indication of the key potential processes controlling the CO_2_-induced and climate-induced carbon uptake/storage [4], [22]. For example, CESM-BGC simulates the smallest sensitivities of land carbon storage to direct CO_2_ effects. As this model includes the CO_2_ fertilization effect constrained by nitrogen limitation compared with other models. Other factors potentially influencing land carbon storage include warming resulting in the changes in land carbon storage, especially at the high latitudes. In the high latitudes and altitudes of Northern Hemisphere, the positive impact of the increasing temperature on land carbon storage has been shown in Figure 7. This is partly due to, in these regions, the enhanced growth caused by the elevated temperatures [49]. Although the intense warming there increases soil organic matter decomposition, soil organic carbon storage has been observed to continue to increase [2]. This change is partly caused by the increase in vegetation productivity, leading to more turnover (litterfall) into the soil [2]. Nevertheless, a qualitative modeled intercomparison of changes in total land carbon storage to increasing temperature, both increasing vegetation storages and soil carbon storage provide useful insights in these temperature-limited regions. Reversely, multi-modeled simulations conformably agree on the negative response of terrestrial ecosystems mainly coming from the reduction in carbon storage in tropics and most Southern Hemisphere.

**Figure 10:**
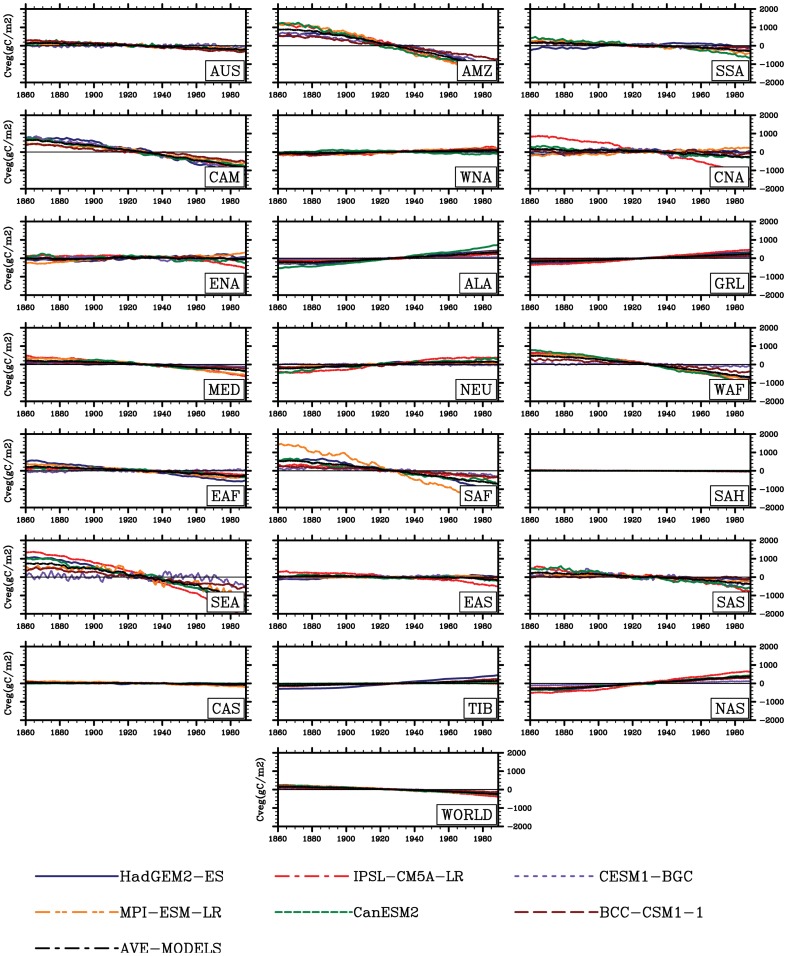
Same as Figure 2, but for vegetation carbon storage (Cveg, gC m^−2^).

**Figure 11:**
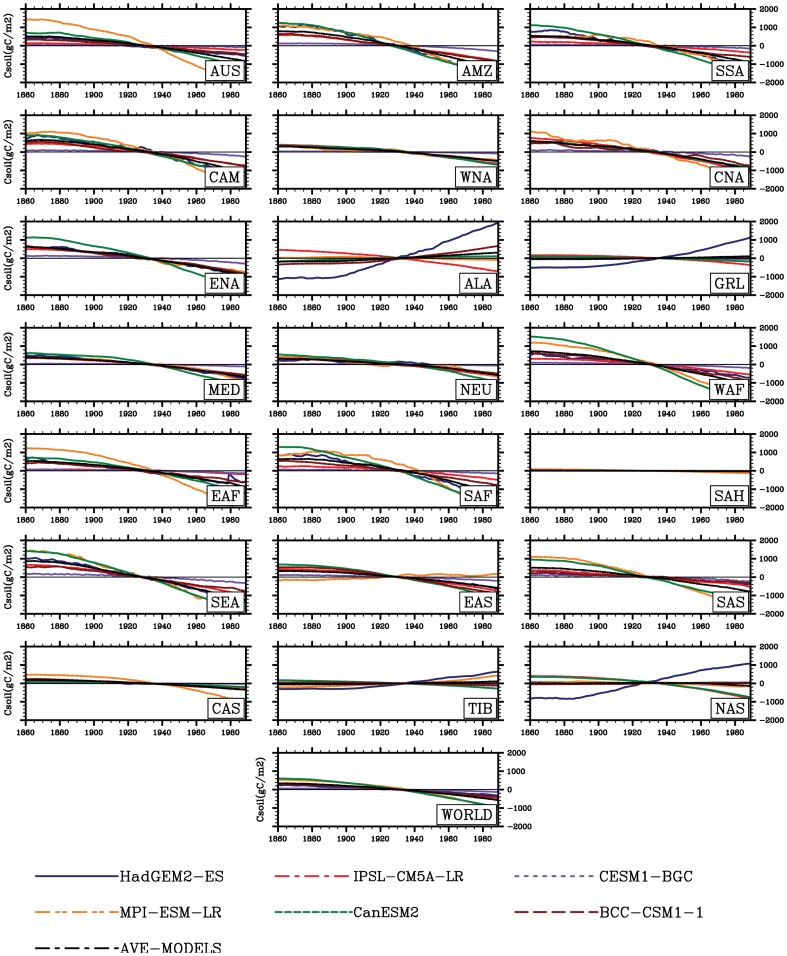
Same as Figure 2, but for soil carbon storage (Csoil, gC m^−2^).

The temperature in this study accounts for a highly important role in the land carbon storage for high latitudes of Northern Hemisphere. Associated with global warming, changes in carbon storage fairly depend on the balance between the input of carbon as the net primary production (NPP) and the loss of carbon as heterotropic respiration (RH) [46]. Over the past two decades, an increase in terrestrial photosynthetic activity has been documented across high latitudes of Northern Hemisphere [39], [50], [51]. NPP has an increase with a rate of about 18.4TgC yr^−2^ (Peng et al unpublished data) in association with the warming in the regions including Alaska, Greenland, northern Europe, Tibet and northern Asia. Thus, accompanied with warming global vegetation growth is significantly elevated in these high latitudes of Northern Hemisphere. Simultaneously, in these regions covered by boreal forest, the lengthened plant growing period has been observed (Linderholm, 2006; Zhu et al., 2012). The longer growing season somewhat contributes such change, based on both the earlier onset of spring [52] and the later ending of autumn [50]. Overall, both experimental and modelling studies suggest greening [53] and increased NPP [2], [50], which infer a upward trend of land carbon storage [2], responding to global warming. This is consistent with our results in high latitudes of Northern Hemisphere. In contrast, in the hot regions such as tropics, the sensitivities of land carbon storage would be generally negative responding to the warming. Such a negative response of carbon storage [54] and reduced carbon sequestration [55] have been also reported in the previous studies. Indeed, unlike in temperature-limited regions, our results suggest that total carbon storage can not be enhanced by the just rising temperature in current high temperature regions (e.g., Amazon basin) (Figures 10–11). This may lead to a net carbon source of the terrestrial ecosystems tend into under the global warming environment. Such a change may be attributed to changes in climatic factors. For example, the tropics exhibit higher increase in temperature and decreases in precipitation and soil moisture as mentioned above. Consequently, reduction in precipitation and soil moisture can result in the diminution in carbon storage and thus such negative response is presented. This is further evident from the 2005 and 2010 drought in Amazon basin [56], [57] leading to reduction in land carbon storage through decreased vegetation productivity and/or increased respiration [16]. In addition, heat and drought can introduce increase in forest dieback [58] and thus a consequence of decreased vegetation production and carbon storage [59]. Compared with change in the vegetation storage produced by this change in vegetation production, a reduction in soil carbon storage can be found due to the decreased litter supply from vegetation (leaves, stems and roots) to soil [60]. On the other hand, the decreased soil storage is commonly generated by the greatly accelerated microbial decomposition with the sequentially rising temperature. Particularly, the larger differences in the responses of total carbon storage to climate change are presented in these regions among the ESMs (e.g., from −13.9 PgCK^−1^ in HadGEM2-ES to −7.5 PgCK^−1^ in CESM1-BGC in Amazon basin), which suggests that the estimation of changes in temperature and precipitation is especially important in evaluating response of carbon cycle of the biosphere. In the same region, the study of Cramer et al. (2001) also suggested the importance of climatic variables for the captured carbon in biomass [13]. Climatic system itself is liable to reduce total carbon storage of terrestrial ecosystems. Hence, improvement in knowledge about it is very important to reduce and even remove uncertainties in sensitivities of land carbon storage at global and regional scales, especially in the areas of around 60°N covered by boreal forest.

Moreover, we compared our results with previous works to clarify the magnitudes and spatial pattern of sensitivities of land carbon storage to rising atmospheric CO_2_ concentration and climate change at both regional and global scales. The used simulations are forced by increasing 1% yr^−1^ until quadruple CO_2_ concentration with/without the carbon-cycle feedback or radiative CO_2_ forcing. Ranges of multi-model sensitivities commonly fall into C^4^MIP model range (sensitivities to increasing CO_2_ between 0.2∼2.8 PgCppm^−1^ and to rising temperature between −177∼−20 PgCK^−1^) [19]. The multi-model positive response to increasing CO_2_ concentrations suggests that the carbon sequestration is improved owning to CO_2_ fertilization effect, especially in broad areas of forests. Such an increase can be explained by the vegetation increased photosynthesis accompanied with the rising atmospheric CO_2_ concentration. For example, the increase of 23.6 gC yr^−1^ (R^2^ = 0.99, P<0.001) in vegetation carbon storage has been shown at the global scale (Figure 5), in association with the increased GPP with a rate of about 5.0 g C m^−2^ yr^−2^(R^2^ = 0.998, P<0.01) (Peng et al unpublished data). For the high latitudes of Northern Hemisphere such as northern Europe and at tropical latitudes such as western Africa, which contain broad areas of forest, great accumulations of carbon into plant biomass appear and the consistent result has be found by Cramer et al.(2001), Sitch et al. (2008) and Ito (2005) [13], [19], [60]. In addition, simulated sensitivities of land carbon storage responding to climate change have large uncertainties. For example, the magnitudes of these sensitivities to temperature in CanESM2 and MPI-ESM-LR are significantly larger than that in CESM-BGC. Compared with the estimates by Arora et al. (2013) [14], the range among multi-model sensitivities to rising CO_2_ is larger, while the range between sensitivities to temperature is smaller. Also such an extent to temperature is smaller than the range assessed by Friedlingstein et al. (2006) [1] and Zickfeld et al. (2011) [23]. Such differences straightforwardly lie in the discrepancy forcing scenario, the used approach and different but plausible representations of the underlying physical and chemical processes [1], [13], [23]. In our study, in all cases sensitivities are calculated based on changes from the last 30-yr results relative to the first 30-yr results of 130-yr simulations. Agreed on simulations from both the multi-model differences in dynamic global vegetation models (DGVMs)[13], [19] and Earth system models (ESMs) [1], [14], the changes of land carbon storage across the terrestrial ecosystem caused by CO_2_ increases of about 563.4 ppm account for about 247% of the changes induced by warming of about 4.3 K. Generally, the magnitude of responses of NPP versus RH to climate change [19], [61] is still debated and strength of dynamical responses of vegetation growth to increasing CO_2_ concentrations is qualitatively different among different models. Such magnitude of NPP has been documented to depend on the local water availability [62]. These changes in water availability depend critically upon uncertain regional aspects of climate change projections and are therefore likely to be another dominant source of uncertainty [14], [19]. Hence the simulated divergences in precipitation and soil moisture can partly contribute to the variances in land carbon storage from the modeled simulations. Overall, these uncertainties can be concluded in the processes: temperature dependent RH [63] and strength of NPP affected by drought and CO_2_ fertilization [62], [64], [65], and captured carbon in the forest aboveground biomass [66], especially in the Amazon forest [67], [68]. Hence, it is urgently needed to pay more attention on key processes (e.g. quantifying the strength of CO_2_ fertilization effect and removing or lessening the uncertainty in climate change) and critical regions. For example, for tropical regions, which play extremely important role in the terrestrial carbon cycle, great uncertainties are even maintained in the competition between the direct CO_2_ effects and climate change due to lacking fully understanding driven mechanism in dynamic biosphere component.

## Conclusion

The results of this study show sensitivities of global and regional land carbon storage to rising atmospheric CO_2_ concentration and climate change, which are directly based on simulations from the CMIP5. Positive impacts of increasing CO_2_ concentration on land carbon storage have been exhibited over the majority of whole terrestrial ecosystems, which are attributed to CO_2_ fertilization effect. At regional scale, the strongest positive impacts mainly occur in broad areas covered by tropical and temperate forests (e.g., Amazon basin, western Africa, southern Asia and southeastern Asia). Great spatial divergence of responses of land carbon storage to warming has been suggested among multi-model simulations. In high latitudes and altitudes, positive effects of increasing temperature are introduced in association with an extended growing season length and enhanced photosynthesis. Current global warming has already accelerated carbon storage loss in most tropics and Southern Hemisphere. This change is partly attributed to local reduction in soil moisture and decline in precipitation. Decreases in land carbon storage of areas including Amazon basin, southern South America, western Africa, eastern Africa, southern Africa and southeastern Asia account for 61.4% simulated by IPSL-CM5A-LR to 97.7% simulated by HadGEM2-ES of the decreases in global land carbon storage responding to the rising temperature. Further, majority of the simulations (≧4/6) agree on the sign of its negative effects of climate change on land carbon storage across low latitudes and Southern Hemisphere. Conversely, across the areas of around 60°N, there is less agreement on effects of climate change among models.

## References

[1] Friedlingstein P, Cox P, Betts R, Bopp L, Von Bloh W, et al (2006) Climate-carbon cycle feedback analysis: Results from the C4MIP model intercomparison. Journal of Climate 19: 3337–3353.

[2] Qian H, Joseph R, Zeng N (2010) Enhanced terrestrial carbon uptake in the northern high latitudes in the 21st century from the coupled carbon cycle climate model intercomparison project model projections. Global Change Biology 16: 641–656.

[3] Randerson JT, Hoffman FM, Thornton PE, Mahowald NM, Lindsay K, et al (2009) Systematic assessment of terrestrial biogeochemistry in coupled climate–carbon models. Global Change Biology 15: 2462–2484.

[4] Schuur EAG, Vogel JG, Crummer KG, Lee H, Sickman JO, et al (2009) The effect of permafrost thaw on old carbon release and net carbon exchange from tundra. Nature 459: 556–559. 19478781 10.1038/nature08031

[5] Yi C, Ricciuto D, Li R, Wolbeck J, Xu X, et al (2010) Climate control of terrestrial carbon exchange across biomes and continents. Environmental Research Letters 5: 034007.

[6] Koven CD, Ringeval B, Friedlingstein P, Ciais P, Cadule P, et al (2011) Permafrost carbon-climate feedbacks accelerate global warming. Proceedings of the National Academy of Sciences 108: 14769–14774. 10.1073/pnas.1103910108 PMC3169129 21852573

[7] Zeng N, Qian H, Munoz E, Iacono R (2004) How strong is carbon cycle-climate feedback under global warming? Geophysical Research Letters 31: L20203.

[8] Cox PM, Pearson D, Booth BB, Friedlingstein P, Huntingford C, et al.. (2013) Sensitivity of tropical carbon to climate change constrained by carbon dioxide variability. Nature, doi:10.1038/nature11882. 10.1038/nature11882 23389447

[9] McGuire AD, Sitch S, Clein JS, Dargaville R, Esser G, et al (2001) Carbon balance of the terrestrial biosphere in the Twentieth Century: Analyses of CO2, climate and land use effects with four process-based ecosystem models. Global Biogeochemical Cycles 15: 183–206.

[10] Zak DR, Pregitzer KS, Kubiske ME, Burton AJ (2011) Forest productivity under elevated CO2 and O3: positive feedbacks to soil N cycling sustain decade-long net primary productivity enhancement by CO2. Ecology letters 14: 1220–1226. 21981597 10.1111/j.1461-0248.2011.01692.x

[11] Peñuelas J, Canadell JG, Ogaya R (2011) Increased water-use efficiency during the 20th century did not translate into enhanced tree growth. Global Ecology and Biogeography 20: 597–608.

[12] Piao S, Ciais P, Friedlingstein P, de Noblet-Ducoudré N, Cadule P, et al.. (2009) Spatiotemporal patterns of terrestrial carbon cycle during the 20th century. Global Biogeochemical Cycles, doi: 10.1029/2008GB003339.

[13] Cramer W, Bondeau A, Woodward FI, Prentice IC, Betts RA, et al (2001) Global response of terrestrial ecosystem structure and function to CO2 and climate change: results from six dynamic global vegetation models. Global change biology 7: 357–373.

[14] Arora VK, Boer GJ, Friedlingstein P, Eby M, Jones CD, et al. (2013) Carbon-concentration and carbon-climate feedbacks in CMIP5 Earth system models. Journal of Climate 26: , 5289–5314.

[15] Hemming D, Betts R, Collins M (2011) Sensitivity and uncertainty of modelled terrestrial net primary productivity to doubled CO_2_ and associated climate change for a relatively large perturbed physics ensemble. Agricultural and Forest Meteorology 170 (15): 79–88.

[16] Heimann M, Reichstein M (2008) Terrestrial ecosystem carbon dynamics and climate feedbacks. Nature 451: 289–292. 18202646 10.1038/nature06591

[17] Boer G, Arora V (2013) Feedbacks in Emission-Driven and Concentration-Driven Global Carbon Budgets. Journal of Climate 26: 3326–3341.

[18] Schimel D, Melillo J, Tian H, McGuire AD, Kicklighter D, et al (2000) Contribution of increasing CO2 and climate to carbon storage by ecosystems in the United States. Science 287: 2004–2006. 10720324 10.1126/science.287.5460.2004

[19] Sitch S, Huntingford C, Gedney N, Levy P, Lomas M, et al (2008) Evaluation of the terrestrial carbon cycle, future plant geography and climate-carbon cycle feedbacks using five Dynamic Global Vegetation Models (DGVMs). Global Change Biology 14: 2015–2039.

[20] Roeckner E, Giorgetta M, Crueger T, Esch M, Pongratz J (2011) Historical and future anthropogenic emission pathways derived from coupled climate–carbon cycle simulations. Climatic Change 105: 91–108.

[21] Arora V, Boer G, Christian J, Curry C, Denman K, et al (2009) The effect of terrestrial photosynthesis down regulation on the twentieth-century carbon budget simulated with the CCCma earth system model. Journal of Climate 22: 6066–6088.

[22] Boer G, Arora V (2010) Geographic aspects of temperature and concentration feedbacks in the carbon budget. Journal of Climate 23: 775–784.

[23] Zickfeld K, Eby M, Matthews HD, Schmittner A, Weaver AJ (2011) Nonlinearity of carbon cycle feedbacks. Journal of Climate 24: 4255–4275.

[24] Arneth A, Harrison SP, Zaehle S, Tsigaridis K, Menon S, et al (2010) Terrestrial biogeochemical feedbacks in the climate system. Nature Geoscience 3: 525–532.

[25] Taylor KE, Stouffer RJ, Meehl GA (2012) An overview of CMIP5 and the experiment design. Bulletin of the American Meteorological Society 93: 485–498.

[26] Peng J, Dong W, Yuan W, Chou J, Zhang Y, et al (2013) Effects of increased CO2 on land water balance from 1850 to 1989. Theoretical and Applied Climatology 111: 483–495.

[27] Peng J, Dan L, Dong W (2014) Are there interactive effects of physiological and radiative forcing produced by increased CO_2_ concentration on changes of land hydrological cycle? Global and Planetary Change 112: 64–78.

[28] Collins W, Bellouin N, Doutriaux-Boucher M, Gedney N, Halloran P, et al (2011) Development and evaluation of an Earth-system model–HadGEM2. Geoscientific Model Development Discussions 4: 997–1062.

[29] Dufresne J-L, Foujols M-A, Denvil S, Caubel A, Marti O, et al.. (2013) Climate change projections using the IPSL-CM5 Earth System Model: from CMIP3 to CMIP5. Climate Dynamics: 1–43.

[30] Girardin MP, Bernier PY, Raulier F, Tardif JC, Conciatori F, et al.. (2011) Testing for a CO2 fertilization effect on growth of Canadian boreal forests. Journal of Geophysical Research doi: 10.1029/2010JG001287.

[31] Raddatz T, Reick C, Knorr W, Kattge J, Roeckner E, et al (2007) Will the tropical land biosphere dominate the climate–carbon cycle feedback during the twenty-first century? Climate Dynamics 29: 565–574.

[32] Todd-Brown K, Randerson J, Post W, Hoffman F, Tarnocai C, et al (2013) Causes of variation in soil carbon simulations from CMIP5 Earth system models and comparison with observations. Biogeosciences 10: 1717–1736.

[33] Wu T, Li W, Ji J, Xin X, Li L, et al.. (2013) Global carbon budgets simulated by the Beijing Climate Center Climate System Model for the last century. Journal of Geophysical Research doi: 10.1002/jgrd.5032.

[34] Brovkin V, Boysen L, Arora V, Boisier J, Cadule P, et al (2013) Effect of anthropogenic land-use and land cover changes on climate and land carbon storage in CMIP5 projections for the 21st century. Journal of Climate 26 (18): 6859–6881.

[35] Krinner G, Viovy N, de Noblet-Ducoudré N, Ogée J, Polcher J, et al.. (2005) A dynamic global vegetation model for studies of the coupled atmosphere-biosphere system. Global Biogeochemical Cycles doi: 10.1029/2003GB002199.

[36] Gent PR, Danabasoglu G, Donner LJ, Holland MM, Hunke EC, et al (2011) The community climate system model version 4. Journal of Climate 24: 4973–4991.

[37] Thornton PE, Doney SC, Lindsay K, Moore JK, Mahowald N, et al (2009) Carbon-nitrogen interactions regulate climate-carbon cycle feedbacks: results from an atmosphere-ocean general circulation model. Biogeosciences 6: 2099–2120.

[38] Thornton PE, Lamarque JF, Rosenbloom NA, Mahowald NM (2007) Influence of carbon-nitrogen cycle coupling on land model response to CO_2_ fertilization and climate variability. Global Biogeochemical Cycles 21 doi: 10.1029/2006GB002868.

[39] Christian J, Arora V, Boer G, Curry C, Zahariev K, et al (2010) The global carbon cycle in the Canadian Earth system model (CanESM1): Preindustrial control simulation. Journal of Geophysical Research 115: G03014.

[40] Arora VK, Boer GJ (2005) A parameterization of leaf phenology for the terrestrial ecosystem component of climate models. Global Change Biology 11: 39–59.

[41] Ji J (1995) A climate-vegetation interaction model: Simulating physical and biological processes at the surface. Journal of Biogeography: 445–451.

[42] Dan L, Ji J, Li Y (2005) Climatic and biological simulations in a two-way coupled atmosphere–biosphere model (CABM). Global and Planetary Change 47: 153–169.

[43] Dan L, Ji J (2007) The surface energy, water, carbon flux and their intercorrelated seasonality in a global climate-vegetation coupled model. Tellus B 59: 425–438.

[44] Giorgi F, Francisco R (2000) Uncertainties in regional climate change prediction: a regional analysis of ensemble simulations with the HADCM2 coupled AOGCM. Climate Dynamics 16: 169–182.

[45] Gao Q, Li Y, Wan Y, Qin X, Jiangcun W, et al (2009) Dynamics of alpine grassland NPP and its response to climate change in Northern Tibet. Climatic change 97: 515–528.

[46] Post WM, King AW, Wullschleger SD (1997) Historical variations in terrestrial biospheric carbon storage. Global Biogeochemical Cycles 11: 99–109.

[47] Piao S, Cui M, Chen A, Wang X, Ciais P, et al (2011) Altitude and temperature dependence of change in the spring vegetation green-up date from 1982 to 2006 in the Qinghai-Xizang Plateau. Agricultural and Forest Meteorology 151: 1599–1608.

[48] Nepstad D, Lefebvre P, Lopes da Silva U, Tomasella J, Schlesinger P, et al (2004) Amazon drought and its implications for forest flammability and tree growth: A basin-wide analysis. Global Change Biology 10: 704–717.

[49] Way DA, Oren R (2010) Differential responses to changes in growth temperature between trees from different functional groups and biomes: a review and synthesis of data. Tree Physiology 30: 669–688. 20368338 10.1093/treephys/tpq015

[50] Zhu W, Tian H, Xu X, Pan Y, Chen G, et al (2012) Extension of the growing season due to delayed autumn over mid and high latitudes in North America during 1982–2006. Global Ecology and Biogeography 21: 260–271.

[51] Myneni RB, Keeling C, Tucker C, Asrar G, Nemani R (1997) Increased plant growth in the northern high latitudes from 1981 to 1991. Nature 386: 698–702.

[52] Christidis N, Stott PA, Brown S, Karoly DJ, Caesar J (2007) Human contribution to the lengthening of the growing season during 1950-99. Journal of Climate 20: 5441–5454.

[53] Alcaraz-Segura D, Chuvieco E, Epstein HE, Kasischke ES, Trishchenko A (2010) Debating the greening vs. browning of the North American boreal forest: differences between satellite datasets. Global Change Biology 16: 760–770.

[54] Fisher JB, Sikka M, Sitch S, Ciais P, Poulter B, et al (2013) African tropical rainforest net carbon dioxide fluxes in the twentieth century. Philosophical Transactions of the Royal Society B: Biological Sciences 368: 20120376. 10.1098/rstb.2012.0376 PMC3720031 23878340

[55] Boyero L, Pearson RG, Gessner MO, Barmuta LA, Ferreira V, et al (2011) A global experiment suggests climate warming will not accelerate litter decomposition in streams but might reduce carbon sequestration. Ecology Letters 14: 289–294. 21299824 10.1111/j.1461-0248.2010.01578.x

[56] Phillips OL, Aragão LE, Lewis SL, Fisher JB, Lloyd J, et al (2009) Drought sensitivity of the Amazon rainforest. Science 323: 1344–1347. 19265020 10.1126/science.1164033

[57] Lewis SL, Brando PM, Phillips OL, van der Heijden GM, Nepstad D (2011) The 2010 amazon drought. Science 331: 554–554. 21292971 10.1126/science.1200807

[58] Bonan GB (2008) Forests and Climate Change: Forcings, Feedbacks, and the Climate Benefits of Forests. Science 320: 1444–1449. 18556546 10.1126/science.1155121

[59] Cox PM, Betts RA, Collins M, Harris PP, Huntingford C, et al (2004) Amazonian forest dieback under climate-carbon cycle projections for the 21st century. Theoretical and Applied Climatology 78: 137–156.

[60] Ito A (2005) Climate-related uncertainties in projections of the twenty-first century terrestrial carbon budget: off-line model experiments using IPCC greenhouse-gas scenarios and AOGCM climate projections. Climate Dynamics 24: 435–448.

[61] Nemani RR, Keeling CD, Hashimoto H, Jolly WM, Piper SC, et al (2003) Climate-driven increases in global terrestrial net primary production from 1982 to 1999. Science 300: 1560–1563. 12791990 10.1126/science.1082750

[62] Zhao M, Running SW (2010) Drought-induced reduction in global terrestrial net primary production from 2000 through 2009. Science 329: 940–943. 20724633 10.1126/science.1192666

[63] Tuomi M, Vanhala P, Karhu K, Fritze H, Liski J (2008) Heterotrophic soil respiration—comparison of different models describing its temperature dependence. Ecological Modelling 211: 182–190.

[64] Berthelot M, Friedlingstein P, Ciais P, Dufresne JL, Monfray P (2005) How uncertainties in future climate change predictions translate into future terrestrial carbon fluxes. Global Change Biology 11: 959–970.

[65] Hickler T, Smith B, Prentice IC, Mjöfors K, Miller P, et al (2008) CO2 fertilization in temperate FACE experiments not representative of boreal and tropical forests. Global Change Biology 14: 1531–1542.

[66] Houghton R (2005) Aboveground forest biomass and the global carbon balance. Global Change Biology 11: 945–958.

[67] Saatchi S, Houghton R, Dos Santos Alvala R, Soares J, Yu Y (2007) Distribution of aboveground live biomass in the Amazon basin. Global Change Biology 13: 816–837.

[68] Martin AR, Thomas SC (2011) A reassessment of carbon content in tropical trees. PLoS One 6: e23533. 21858157 10.1371/journal.pone.0023533 PMC3157388

